# Unveiling essential host genes and keystone microorganisms of the olive tree holobiont linked to Verticillium wilt tolerance

**DOI:** 10.1186/s40168-025-02216-5

**Published:** 2025-11-26

**Authors:** Antonio J. Fernández-González, Alicia Serrano, Francisco Luque, Manuel Fernández-López, Jesús Mercado-Blanco

**Affiliations:** 1 Department of Soil and Plant Microbiology, Microbiology of Agroforestry Ecosystems Group, Estación Experimental del Zaidín, CSIC, Granada, 18008 Spain; 2 Department of Experimental Biology, University Institute of Research On Olive Grove and Olive Oils, University of Jaén, Jaén, 23071 Spain

**Keywords:** Microbiome engineering, *Actinophytocola*, *Nocardia*, Plant defense response genes, Belowground microbiota, Co-occurrence network

## Abstract

**Background:**

The plant holobiont concept emphasizes the critical role of the microbiome in host plant health and resilience. Microbial communities have been shown to enhance plant resistance to abiotic stresses, such as drought and salinity, and to mitigate the impact of phytopathogens. Traditional microbiome engineering approaches face challenges due to the complexity of microbial interactions. To overcome these limitations, recent advances in transcriptomics and metataxonomics analyses enable the identification of microbiome-associated phenotypes, co-occurrence networks, and key host genes-microbiome interactions. We present a novel framework combining co-occurrence network analyses and transcriptome-microbiota correlations to identify keystone belowground microorganisms and host genes potentially involved in olive (*Olea europaea* L.) tolerance to Verticillium wilt, a devastating disease caused by the soil-borne, fungal vascular pathogen *Verticillium dahliae* Kleb. Our approach aims to identify microbiome-regulating host genes and keystone bacteria and fungi that could be instrumental as genetic and microbiological markers in olive breeding programs.

**Results:**

In the root endosphere, cultivars qualified as tolerant to Verticillium wilt of olive (VWO) exhibited an enrichment of the bacterial genera *Actinophytocola*, *Kibdelosporangium* and *Nocardia*. Keystone taxa analyses revealed clearly different profiles when comparing the microbial co-occurrence networks of the VWO-tolerant genotypes with those varieties described as susceptible to *V. dahliae*. Thus, tolerant cultivars harbored bacteria predominantly displaying negative interactions with the mycobiome. In contrast, VWO-susceptible cultivars displayed microbial hubs with positive fungal correlations. Transcriptomic analyses of olive roots identified 1,143 differentially expressed genes (DEGs), with 309 upregulated genes in tolerant cultivars, highlighting biological processes like defense response, carbohydrate metabolism, and amino acid transport. Key microbial taxa (*Actinophytocola*, *Kibdelosporangium*, *Nocardia*, *Aquabispora*, and *Fusarium*) strongly correlated with DEGs associated with plant defense.

**Conclusions:**

Keystone microbial taxa, particularly *Actinophytocola* and *Nocardia*, are proposed to play an important role against *V. dahliae* within the indigenous olive root microbiota under natural conditions. Moreover, our findings underscore the importance of studying keystone taxa along with essential host plant genes to holistically understand plant-microbiota interactions and explore their potential in disease management. This integrative approach provides insights into the complex dialogue taking place between the host plant and its microbiota, offering potential targets for microbiome engineering to enhance olive resilience against VWO.

Video Abstract

**Supplementary Information:**

The online version contains supplementary material available at 10.1186/s40168-025-02216-5.

## Background

The importance of the microbiome in the host plant fitness has been highlighted within the framework of the holobiont concept [1, 2]. Indeed, several studies have demonstrated enhanced resistance to abiotic stresses, such as salinity and drought, in plants of the same genotype but harboring different microbiota [3, 4]. Similarly, a correlation has been observed between the enrichment of certain members of the plant microbial community and increased resistance to diseases caused by phytopathogens [5, 6].

In this context, novel strategies have emerged to harness the beneficial properties of the microbial component within the holobiont through microbiome engineering [7, 8]. Traditionally, the most widely implemented approach is the inoculation with one or more (i.e., microbial consortia or the so-called synthetic communities, SynCom) microorganisms previously selected (bottom-up method) because of the beneficial traits provided to the target plant (e.g., improved growth, nutrient assimilation, or activation of the plant immune system). This strategy is supported by relatively easy procedures of isolation and growth under laboratory conditions of the selected microorganisms. However, some studies have also demonstrated the utility of an alternative strategy (top-down method) focused on in situ microbiome modification through the directed evolution of the microbial community [9].

While both approaches have great potential, they also pose significant limitations [10]. The top-down method is constrained to plants with short life cycles, since multiple generations are required to select for the desired holobiont trait, or directed evolution is needed in a natural substrate (e.g., soil) without the host [4]. Conversely, the bottom-up method may demand time-consuming work to isolate microorganisms, perform in vitro antagonism assays, and carry out greenhouse trials before field applications. Unfortunately, successful outcomes are only evident at the final stages, and many efforts fail due to our limited understanding of the interactions occurring between the introduced inocula and the pre-existing microbial community in the target niche [11].

To address these shortcomings, recent studies focus on designing microbiome engineering strategies that combine the strengths of both methods to maximize their benefits [12]. Furthermore, research over the years has highlighted the pivotal role of keystone taxa in enhancing the holobiont fitness through its microbiome [1, 12, 13]. Current technologies already enable, or will soon allow, correlating a specific holobiont phenotype with its microbiome. This can be achieved by microbiome-associated phenotypes [14], cotranscriptomic studies, combining host and microbiome transcriptomics [2], or through integrating transcriptomic data with microbiota (metataxonomics) analyses [15, 16].

However, it is crucial not only to study plant-microbiome interactions but also to identify the microbe-microbe correlations co-occurring among the constituents of a given microbiome. While the methods available for inferring such interactions are subjected to biases and require careful interpretation [17, 18], several studies have demonstrated that co-occurrence network analyses are valuable and complementary tools for identifying key microorganisms. For instance, Zheng and collaborators showed that enriching keystone taxa within the soil microbiome through inoculation accelerated organic matter decomposition and resulted in a more stable and lasting microbiome modification [19]. Lin and co-workers used a different approach by adding prebiotics to enhance keystone taxa populations when microbial isolation was not feasible. This strategy successfully altered the microbiome in the desired direction [20].

In this present study, we propose the combination of co-occurrence network methods and transcriptome-microbiota correlation analyses in order to identify key host genes and keystone microorganisms potentially linked to tolerance to Verticillium wilt of olive (VWO). This disease, caused by the soil-borne fungus *Verticillium dahliae* Kleb., is known for decades as one of the most serious menaces for olive groves, particularly in the Mediterranean Basin. To date, no single control measure has been proven effective to eradicate or substantially mitigate the impact of VWO. Therefore, an integrated disease management strategy is highly recommended, with emphasis in preventive actions [21].

The premises on which our starting hypothesis is based are: i) the olive microbiome is strongly influenced by the host phenotype; ii) keystone taxa are essential for microbiome stability and effective plant-microbiota communication, and iii) some of these taxa are critical for plant health and successful defense against *V. dahliae*. Therefore, we aimed to: i) assess whether the olive belowground microbiota assembles differently in VWO-tolerant and VWO-susceptible genotypes; ii) identify differences in keystone taxa and transcriptomic profiles between tolerant and susceptible olive cultivars; and iii) unveil whether certain enriched microorganisms are positively correlated with the expression of defense-related genes against VWO. To achieve these aims we focused on healthy olive trees with markedly contrasting phenotypes regarding their tolerance or susceptibility to *V. dahliae*.

## Materials and methods

### Data collection and starting considerations

A data mining strategy was used in our study. Olive belowground microbiota datasets were earlier generated by high-throughput amplicon sequencing [22], while the transcriptomes of olive root tissues were obtained by RNA-Seq [23]. Metataxonomics and transcriptomics datasets originated from the same olive trees. Roots were sampled in the same sampling event (spring 2017) carried out in the World Olive Germplasm Collection (WOGC) located at The Andalusian Institute of Agricultural and Fisheries Research and Training (IFAPA, Cordoba, Spain). Details on the olive orchard, olive genotypes and their geographical origins, experimental designs, soil and root sampling procedures, and DNA NGS and RNA-seq have been detailed elsewhere [22, 23]. We would like to emphasize that all olive trees (36 different genotypes originating from nine countries) were grown in the same orchard (i.e., subjected to the same pedological, climatic and management conditions). The same belowground microbiota raw data reported by Fernández-González et al. [22] were used but a new analysis has been carried out (see below). A new normalization analysis of the olive root RNA-Seq dataset earlier published by Ramírez-Tejero et al. [23], and its correlation with the endophytic microbiota are also described below.

### Olive genotype selection

Serrano et al. [24] recently published the most comprehensive study on tolerance/susceptibility of olive cultivars to VWO. Based on the screening and classification performed by these authors, we aimed to confront the available transcriptomic datasets (root level) [23] of those cultivars displaying the most extreme phenotypes (i.e., either highly resistant or extremely susceptible to VWO [24]). Then, we examined whether these phenotypes correlated with a differential assembly of the root-associated microbiome present in the selected cultivars (cv.). Nevertheless, high quality data [22] were available only from one highly resistant genotype (‘Frantoio’) and five resistant ones (‘Manzanillera de Huércal-Overa’, ‘Maarri’, ‘Uslu’, ‘Dokkar’ and ‘Koroneiki’), according to the classification by Serrano et al. [24]. Eventually, data from cv. ‘Dokkar’ were discarded due to the lack of replicates in the bacterial library. Thus, the VWO-tolerant group of olive cultivars was made up of five genotypes. The number of VWO-susceptible genotypes is much higher, as widely reported in the literature. Thus, RNA-Seq data were available [23] from the nine extremely susceptible genotypes classified by Serrano et al. [24]. However, in order to have a more statistically balanced study, five genotypes were eventually selected. The main selection criterion was to include at least one representative of some of the countries of origin present in the WOGC. Thus, the VWO-susceptible group resulted in the following cultivars: ‘Abbadi Abou Gabra-842’, ‘Chemlal de Kabylie’, ‘Jabali’, ‘Mastoidis’ and ‘Temprano’. The cv. ‘Picual’ was also included in this group even though it was qualified as VWO-susceptible instead of VWO-extremely susceptible [24]. Two major reasons supported this decision: i) ‘Picual’ has been frequently used as model genotype for susceptibility to VWO in previous studies [25–27]; and ii) it is extensively cultivated in many olive cropping areas, particularly in Andalusia (Southern Spain), the largest olive oil producer region worldwide [28].

### Microbiota data processing

The original raw data [22] were re-processed through our tutorial developed by Wentzien et al. [29]. This workflow allows the raw data to be analyzed on a single platform and programming language in an intuitive and computationally friendly way. In addition, it makes use of the most current and frequently used tools in metataxonomics. For this reason, in the present study we identified Amplicon Sequence Variants (ASVs) instead of grouping the sequences into Operational Taxonomic Units (OTUs). It is worth mentioning that the root DNAs of the cultivars ‘Frantoio’ and ‘Picual’ were re-sequenced in order to have an optimal number of reads to be included in this present study. These newly-generated raw data have been incorporated in the previously published BioProject PRJNA498945 [22]. Briefly, reads filtering and ASVs inference was performed with DADA2 [30]. Then, the bacterial and fungal libraries were classified with the Ribosomal Database Project II, training set v. 18 [31] and the UNITE v. 9.0 dynamic database [32], respectively. Finally, those ASVs with a relative abundance lower than 0.005% were eliminated together with those that belonged to the host DNA or remained unclassified at the phylum level (the final ASV and the samples metadata tables are available in Additional File 1).

Following the tutorial mentioned above, alpha and beta diversity analyses were performed, and microbial profiles of the main genera were generated. To produce clear taxonomic profile plots, genera with a relative abundance ≥ 1% in at least one of the groups were considered the main genera, but only those with relative abundance ≥ 2% in at least one of the groups were represented. As an exception, *Nocardia*, *Metabacillus* and *Rhizophagus* were included as they showed > 1% relative abundance and were significantly enriched in one group. Finally, differentially abundant genera were identified in both the bacterial and fungal libraries. The root endosphere and the rhizosphere compartments were considered independently in the entire process. The comparison of the alpha diversity indices (observed richness, Shannon, inverse of Simpson and evenness) was carried out using Wilcoxon tests in the rarefied data. These data showed homogeneity of variances (Levene's test), but not all indices showed normality (Shapiro test). In the case of beta diversity, the microbiota of the two groups of olive cultivars (VWO-tolerant and VWO-susceptible) were compared using PERMANOVA. Bray–Curtis and (un)weighted UniFrac (only the first in the case of the mycobiome) of TMM normalized data were used for dissimilarity/distance matrices calculation. In addition, homoscedasticity was assessed using BETADISPER. The principal coordinates analysis (PCoA) and non-metric multidimensional scaling (NMDS) plots were visualized using the *plot*_*ordination* tool of the phyloseq package [33]. Finally, to evaluate which genera showed differential abundance between the two groups of olive cultivars, those with statistically significant uncorrected *p*-values ​​were selected. In fact, no genus showed a statistically significant differential abundance after false discovery rate (FDR) correction in the two most reliable methods according to Nearing et al. [34]. These methods, ANCOM-BC2 [35] and ALDEx2 [36], consider the inherent compositionality and sparsity of this type of data, but tend to have a higher false negative rate than other less accurate methods [34].

### Analysis of differentially expressed genes

The olive root transcriptomes published by Ramírez-Tejero et al. [23] were used for the gene expression analysis. Specifically, the raw data corresponding to five VWO-tolerant olive cultivars (‘Frantoio’, ‘Koroneiki’, ‘Maarri’, ‘Manzanillera de Huércal-Overa’, and ‘Uslu’) and six VWO-susceptible ones (‘Abbadi Abou Gabra-842’, ‘Chemlal del Kabylie’, ‘Jabali’, ‘Mastoidis’, ‘Picual’, and ‘Temprano’) were downloaded.

For transcriptomic analysis, STAR v2.7 [37] was used to align the raw reads to the reference genome of ‘Picual’ [38]. For this step, the genome was required in FASTA format and its annotated version in GTF format. Both formats are available in OliveTreeDB [39]. After alignment, the BAM files were sorted by name using Samtools v1.16 [40] for input into featureCounts v1.16.1 for counting the mapped transcripts [41]. Subsequently, after removing cv. ‘Maarri’ from the VWO-tolerant group (see biological explanation in the Results section), the differentially expressed genes (DEGs) between tolerant and susceptible cultivars were identified. To this end, DESeq2 v1.38.3 [42] was applied considering *p*-values adjusted (FDR) ≤ 0.05 and fold change (FC) ≥ 2 for overexpressed genes, and FC ≤ –2 for repressed genes, using the VWO-tolerant group as reference*.* The description of the most representative biological processes in the set of overexpressed genes in the tolerant and susceptible groups was performed by gene enrichment analysis using ShinyGO v0.8 web tool [43]. The program returns the GO terms of the most representative biological processes based on an FDR ≤ 0.05 and a fold enrichment value. ShinyGO v0.8 automatically calculates this fold enrichment parameter based on the number of genes in the DEGs set compared to the set provided as background, which was the complete transcriptome of' Picual' (81,484 genes).

Differentially expressed genes were re-annotated using the Sma3s v.2 tool [44] and the UniProt plant protein database combining the resources of the Swiss-Prot and TrEMBL (version 2024_02). The updated gene annotation was used for subsequent interaction analysis between DEGs and ASVs.

### Co-occurrence network construction and visualization

After removing cv. ‘Maarri’ from the VWO-tolerant group (see biological explanation in the Results section), four molecular ecological networks were generated through the MENAP web tool [45]. To do this, the ASV tables of bacteria and fungi from each group of olive trees (tolerant and susceptible) were merged for each compartment (root endosphere and rhizosphere) separately. In the case of the two networks of the VWO-tolerant group, the ASVs with a prevalence of 50% were retained for their construction (*n* = 12, 4 genotypes × 3 replicates). However, for the VWO-susceptible group (*n* = 18, 6 genotypes × 3 replicates), prevalences of 39% in the endosphere (threshold = 7) and 44% in the rhizosphere (threshold = 8) were used. To analyze the correlation between the endophytic microbiota and the transcriptomic profiles of the roots, a network was built considering all olive genotypes. Therefore, the bacterial and fungal ASV tables were merged with the DEGs table selected in the previous section. In this case, DEG count values without normalizing were used. For this correlation network between ASVs and DEGs, a prevalence of 50% was used (*n* = 20, 10 genotypes × 2 replicates). It must be emphasized that the similarity matrices of the five co-occurrence networks (two microbe-microbe networks, endosphere and rhizosphere, per VWO-tolerant and VWO-susceptible groups, plus transcriptome-microbiota network) were computed with the second Spearman method offered by the website. The choice of this correlation method was based on the recommendation of the developers for our type of data and which we have already used in previous works [15, 46]. To establish the Spearman's *Rho* threshold, the lowest value was always chosen as long as it showed a *P* > 0.05 in the Random Matrix Theory (RMT) test when comparing the regression curve of our data with a Poisson distribution. Thus, in order to compare the VWO-tolerant *vs.* the VWO-susceptible groups in both the rhizosphere and the endosphere, similar thresholds (or with a difference ≤ 0.05) were chosen. In fact, the same values ​​were obtained for the two networks in the endosphere (*Rho* = 0.73), and very similar values ​​for the rhizosphere (*Rho* = 0.88 and 0.83 for VWO-tolerant and VWO-susceptible cultivars, respectively). However, for the transcriptome-microbiota correlation network, a threshold value with *P* > 0.05 was not obtained. Therefore, *Rho* = 0.73 was decided as the most appropriate value for the endophyte microbe-microbe correlation networks. Finally, to visualize the co-occurrence networks, the Cytoscape v. 3.10.2 [47] was used.

### Host transcriptome-microbiota correlation

The DEGs obtained by comparing VWO-tolerant and VWO- susceptible groups by DESeq2 with the above-described parameters were filtered with the RMT method. Thus, this study focused mainly on those DEGs with statistically significant differences between the two groups of genotypes with the most extreme VWO tolerance/susceptibility profiles, and that showed a strong correlation (−0.73 ≤ Spearman’s *Rho* ≥ 0.73) with some microbial endophytes.

## Results

### Beta diversity as key index in the selection of extreme phenotypes for VWO tolerance

The initial comparative study of the belowground microbiota of the VWO-tolerant and VWO-susceptible groups showed no differences in terms of alpha diversity. In contrast, statistically significant differences were observed when assessing beta diversity, although only by Bray–Curtis (See Table S1, Additional File 2). Moreover, this method did not only unravel the difference between the two groups but also allowed to discriminate a genotype that could be assigned with some uncertainty in the study of Serrano and co-workers [24]. Indeed, cv. ‘Maarri’ (See Figure S1, Additional File 2) was previously classified as resistant to VWO by these authors. However, a closer examination of their data (mainly, a low level of FHP = Final healthy plants incidence) could suggest a lower level of tolerance compared to that displayed by the other four genotypes qualified as VWO-tolerant. In fact, ‘Maarri’ also showed a transcriptomic profile more similar to that of the extremely susceptible genotypes (See Figure S2, Additional File 2), supporting the results of the beta diversity. Therefore, taking into account this outcome and for further analyses in this study, the VWO-tolerant group was reduced to cultivars ‘Frantoio’, ‘Manzanillera de Huércal-Overa’, ‘Uslu’ and ‘Koroneiki’, whereas the VWO-susceptible group remained as originally selected (‘Abbadi Abou Gabra-842’, ‘Chemlal de Kabylie’, ‘Jabali’, ‘Mastoidis’, ‘Picual’ and ‘Temprano’).

### Revisiting the olive belowground microbial diversity without ‘Maarri’

Interestingly, when cv. ‘Maarri’ was excluded from the analysis, greater evenness was observed in the endophytic bacteria of the VWO-susceptible group compared to that of the VWO-tolerant group. In contrast, an increase in richness and diversity, but not evenness, was observed in the case of endophytic fungi (Table 1). However, alpha diversity of the rhizosphere microbiota was similar in both groups. Regarding beta diversity, differences between susceptible and tolerant cultivars were found in both compartments (rhizosphere and endosphere) and for the bacteriota and the mycobiota. It is worth noting that, although statistically significant, the tolerance/susceptibility to VWO factor had a relatively moderate weight in the belowground microbiota assembly (< 9% of the explained variance, see R^2^ in Table 1).

**Table 1:** Alpha and beta diversity of olive groups tolerant and susceptible to VWO without cultivar ‘Maarri’

		Bacteria			
	Factor	Observed richness	Shannon	Inv. Simpson*	Evenness
Endosphere	Tolerant	59.75 ± 27.23	2.93 ± 0.61	12.88 ± 9.26	***0.73* ± *0.09***
	Susceptible	69.17 ± 38.88	3.26 ± 0.77	19.40 ± 12.51	***0.79* ± *0.12***
Rhizosphere	Tolerant	622.92 ± 92.61	5.48 ± 0.40	84.2 ± 73.08	0.85 ± 0.05
	Susceptible	628.00 ± 104.95	5.62 ± 0.38	124.59 ± 78.12	0.87 ± 0.05
			PERMANOVA		PERMDISP2
		Distance/dissimilarity metric	R^2^	*P*	*P*
Endosphere		Bray–Curtis	0.0809	***0.002***	***0.041***
		UniFrac	0.0383	0.287	0.567
		weighted UniFrac	0.0894	***0.040***	0.203
Rhizosphere		Bray–Curtis	0.0815	***0.002***	***0.024***
		UniFrac	0.0868	***0.001***	0.063
		weighted UniFrac	0.0564	0.107	0.160
		Fungi			
	Factor	Observed richness	Shannon	Inv. Simpson*	Evenness
Endosphere	Tolerant	***28.00* ± *11.60***	***2.00* ± *0.53***	5.46 ± 1.92	0.61 ± 0.14
	Susceptible	***45.33* ± *17.75***	***2.51* ± *0.63***	8.97 ± 5.44	0.66 ± 0.11
Rhizosphere	Tolerant	115.50 ± 11.24	3.57 ± 0.30	18.77 ± 8.48	0.75 ± 0.06
	Susceptible	116.28 ± 23.12	3.60 ± 0.35	19.19 ± 8.55	0.76 ± 0.06
			PERMANOVA		PERMDISP2
		Distance/dissimilarity metric	R^2^	*P*	*P*
Endosphere		Bray–Curtis	0.0725	***0.001***	0.069
Rhizosphere		Bray–Curtis	0.0569	***0.015***	0.100

The factor to be compared in the different biodiversity indices was tolerance versus susceptibility to VWO. Statistically significant differences in alpha-diversity and *p*-values in beta-diversity are highlighted in bold type and italics. *Inv. Simpson = Inverse of Simpson

### Marked differences in the main endophytic microbiota

**Fig. 1:**
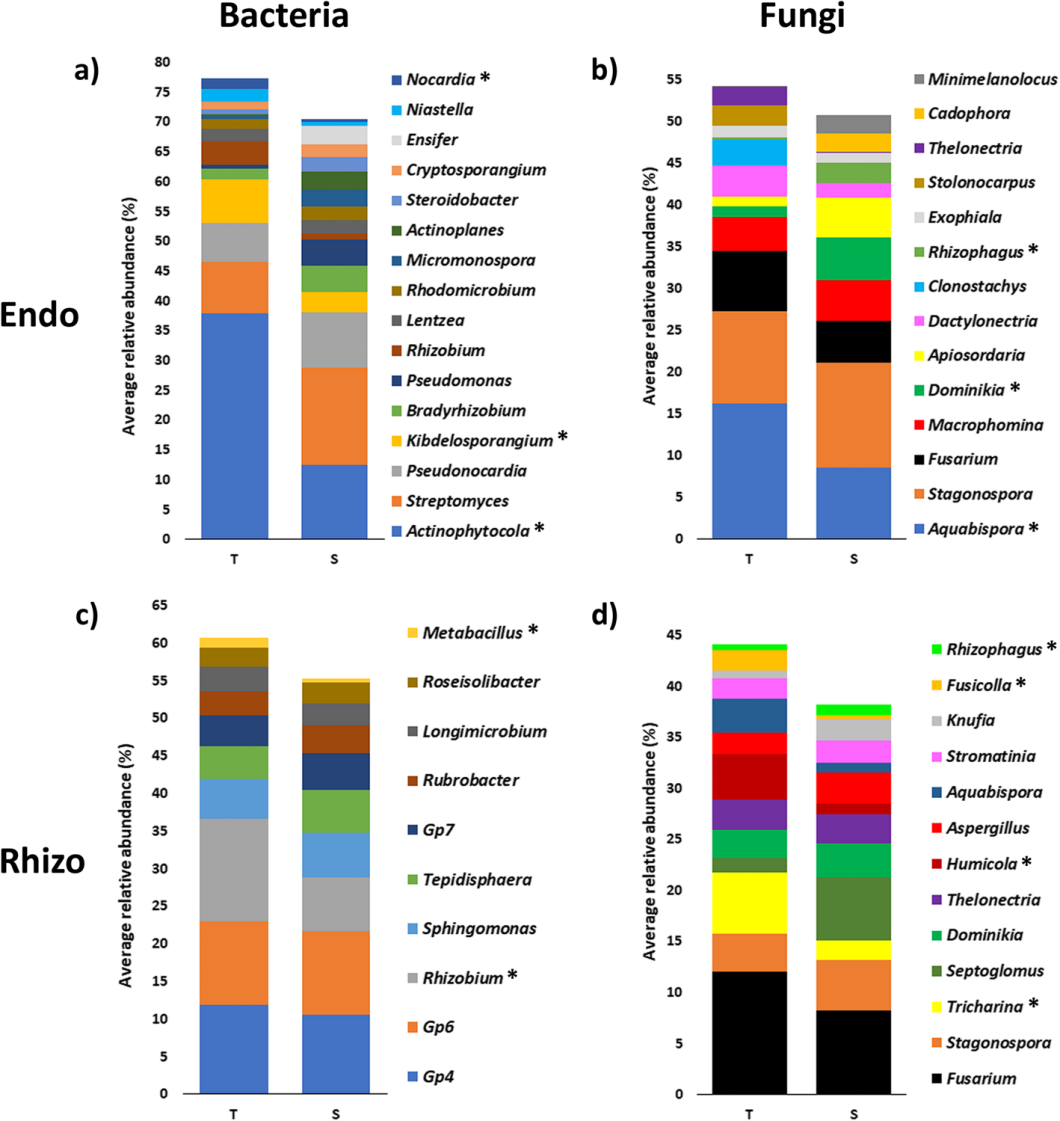
Taxonomic profiles at the genus level of olive cultivars belowground microbiomes. Bacterial (**a** and **c**) and fungal (**b** and **d**) communities of VWO-tolerant (T) and VWO-susceptible (S) cultivars from endosphere and rhizosphere, respectively. Endo = endosphere, Rhizo = rhizosphere. Genera with statistically significant differences between both groups were highlighted with an asterisk

An enrichment of *Actinophytocola*, *Kibdelosporangium*, *Nocardia*, *Rhizobium* and *Niastella* was observed in the root endosphere bacteriome of the VWO-tolerant group, although statistically significant only for the first three genera. In contrast, most of the main genera, headed by *Streptomyces*, *Pseudonocardia*, *Bradyrhizobium* and *Pseudomonas*, were more enriched in the root endosphere of the VWO-susceptible group, although without statistical support (Fig. 1a). Main genera in the rhizosphere, however, showed rather similar profiles in both groups, with only two genera (*Rhizobium* and *Metabacillus*) significantly more enriched in the VWO-tolerant group (Fig. 1c).

Concerning the mycobiome, substantial differences were observed both inside and outside the roots. The genera *Aquabispora* and *Neocosmospora* were enriched in the VWO-tolerant group and the arbuscular mycorrhizal fungi (AMF) of the genera *Dominikia* and *Rhizophagus* were enriched in the VWO-susceptible group within the roots (Fig. 1b). In the rhizosphere, *Tricharina*, *Humicola* and *Fusicolla* showed a statistically significant enrichment in the VWO-tolerant group. *Rhizophagus* was the only main genus that showed a statistically significant depletion in this group, although *Septoglomus* and *Dominikia* (all of them AMF) showed the same trend (Fig. 1d). Significant changes were also observed in minor genera of the fungal community, mainly in the rhizosphere. Indeed, depletion in *Minimelanolocus* and *Entoloma* and increase in *Mortierella*, *Hormonema*, *Malassezia* and *Trichoderma* were observed in the VWO-tolerant cultivars (See Additional File 3). Finally, *Fusarium* was the first and third most abundant genus in the rhizosphere and the endosphere, respectively. The relative abundance of this genus tended to increase in the VWO-tolerant group, although no statistically significant differences were observed between the two groups of cultivars (Fig. 1b and d).

### Keystone microbial taxa were noticeably different depending on the VWO tolerance/susceptibility of the cultivar

**Fig. 2:**
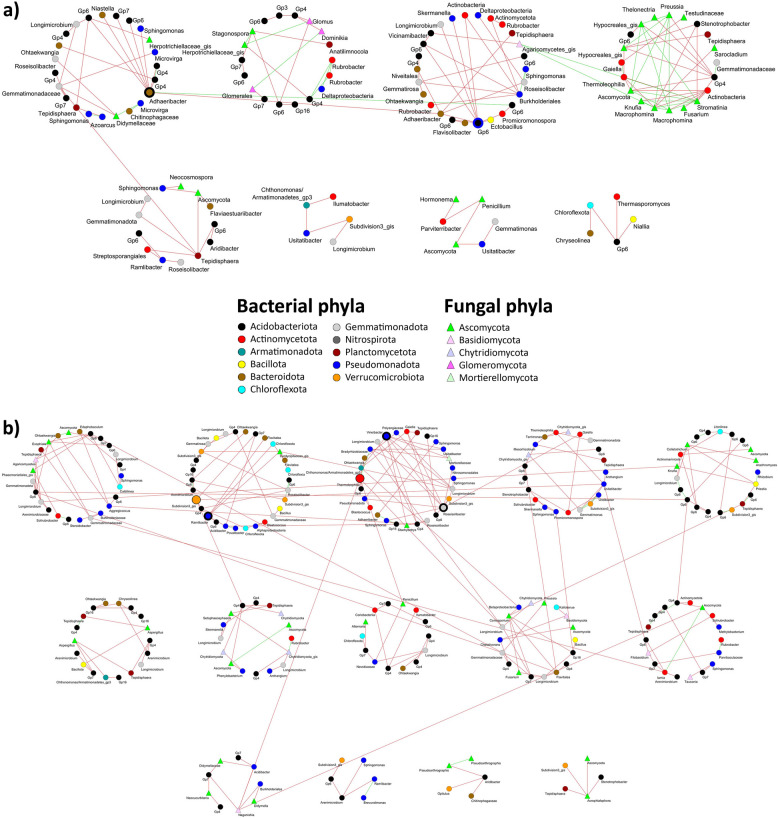
Co-occurrence networks of VWO-susceptible (**a**) and VWO-tolerant (**b**) from rhizosphere microbial communities. Keystone ASVs are highlighted by larger sizes and thicker border: Module hub with border thickness = 20 and connector with border thickness = 10, both with node size = 50. The rest of ASVs were drawn with border thickness = 1 and node size = 30. Keystone ASVs from Acidobacteria (black nodes) were drawn with blue border

**Fig. 3:**
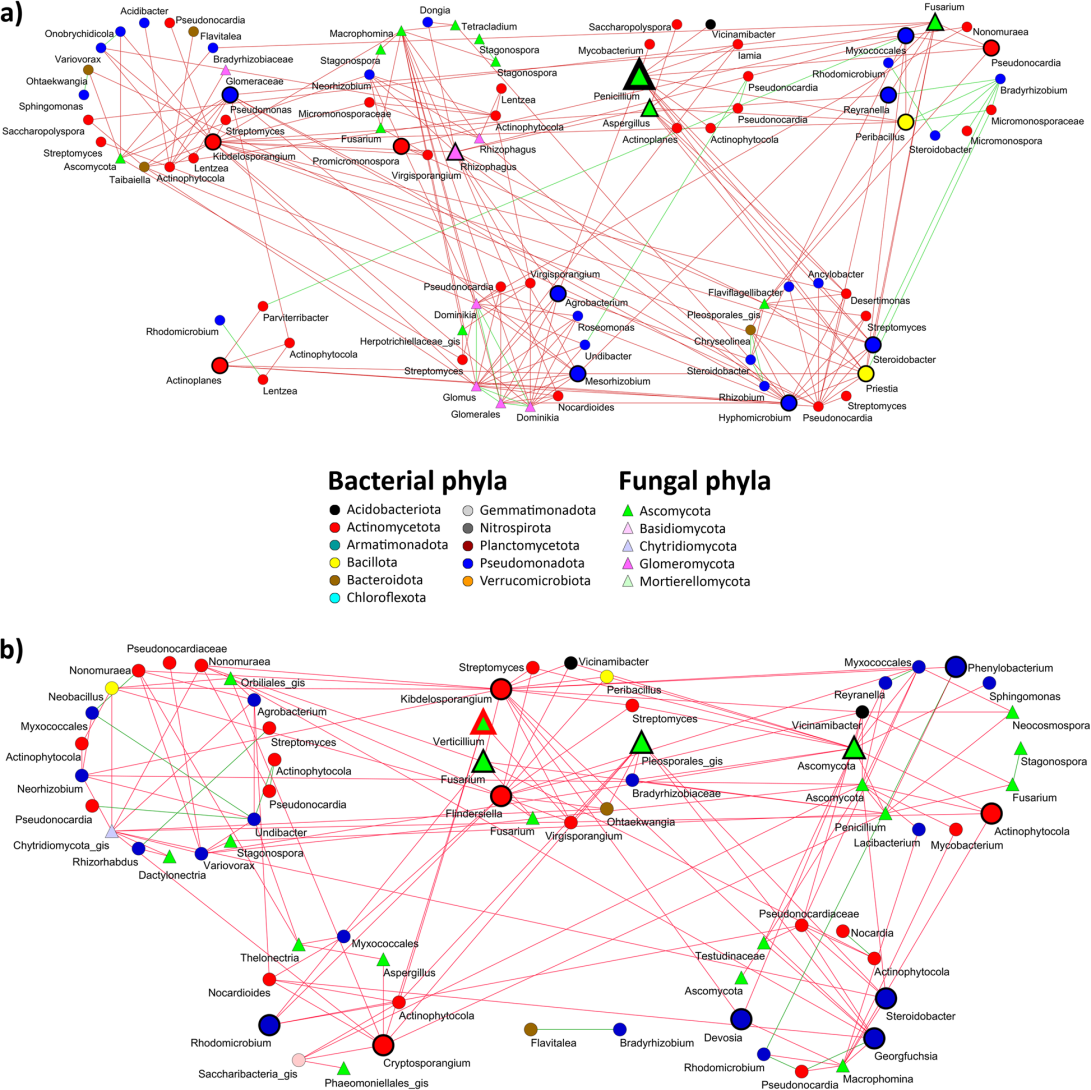
Co-occurrence networks of VWO-susceptible (**a**) and VWO-tolerant (**b**) from endosphere microbial communities. Keystone ASVs are highlighted by larger sizes and thicker border: Network hub with node size = 90 and border thickness = 50, module hub with border thickness = 20 and connector with border thickness = 10, both with node size = 50. The rest of ASVs were drawn with border thickness = 1 and node size = 30. The ASV belonging to *Verticillium* was highlighted with a thick red border

Some global topological properties (avgK and M) of the co-occurrence networks were similar for the rhizosphere communities of the VWO-tolerant and VWO-susceptible groups. However, a higher degree of intramodular connectivity was observed in the network of the VWO-susceptible group (higher Transitivity and avgCC). This could contribute to the decrease in average geodesic distance (GD) shown by this network (Table 2). Besides, and more importantly, these two networks showed very different keystone taxa profiles, both quantitatively and qualitatively. The VWO-susceptible group showed two bacterial module hubs (*Adhaeribacter* and *Gp6*) with negative connections to other bacterial members of their same modules. In addition, one ASV of the fungal genus *Stromatinia* was found to be highly connected, most of its links being positive with a large number of fungi of its own module (Fig. 2a). However, three module hubs (*Ramlibacter*, *Vineibacter* and *Roseisolibacter*) and two connectors (Subdivision3_gis and *Thermoleophilia*) were identified in the VWO-tolerant group, all of them clustered in the two largest modules and showing 100% of negative links (Fig. 2b). Regarding the root endosphere microbiota, the most relevant findings were the identification of (i) only connectors in keystone taxa from the VWO-tolerant group (*Fusarium*, *Pleosporales*_gis, *Kibdelosporangium*, *Flindersiella*, Ascomycota, *Phenylobacterium*, *Actinophytocola* (ASV00100), *Devosia*, *Georgfuchsia*, *Steroidobacter*, *Rhodomicrobium* and *Cryptosporangium*), and (ii) three actinobacterial ASVs (*Actinophytocola* (ASV00039), *Virgisporangium*, and the same ASV belonging to *Cryptosporangium* that appears as keystone) negatively correlated with one ASV annotated as *Verticillium*. It is important to note that the ASV00039 annotated as *Actinophytocola* was neither the most abundant (ASV00005) nor the keystone (ASV00100). However, it is worth mentioning that ASV00100 was negatively correlated with several fungal ASVs as well (Fig. 3b). It was also remarkable the presence of ASVs from the three genera significantly enriched in the VWO-tolerant group (*Actinophytocola*, with one ASV negatively interacting with *Verticillium*, *Kibdelosporangium*, a keystone ASV, and *Nocardia*). The profile of keystone taxa in the VWO-susceptible group was very different, with the genus *Penicillium* as network hub and 16 connectors of diverse taxonomic phyla (Ascomycota, Glomeromycota, Pseudomonadota, Actinomycetota and Bacillota). Only ASV00105 belonging to the actinobacterial genus *Kibdelosporangium* (connector) played a similar key role in both networks (see “nodes” sheets, Additional File 4, for detailed parameters of each node). It is worth noting the large number of ASVs belonging to arbuscular mycorrhizal fungi found in this co-occurrence network, and with a node of the genus *Rhizophagus* as a keystone. Surprisingly, no correlation was observed between the ASV of *Verticillium* and any microbial endophyte in the co-occurrence network of this group (Fig. 3a).

**Table 2:** The major topological properties of the microbe-microbe co-occurrence networks

		Total nodes	Total links	R^2^	St	avgK	avgCC	GD	Trans	M
Endo	T	68	150	0.743	0.73	4.412	***0.045***	***2.987***	***0.028***	***0.403 (6)***
	S	85	196	0.813	0.73	4.612	***0.091***	***3.271***	***0.107***	***0.447 (7)***
Rhizo	T	316	315	0.860	0.88	1.994	***0.005***	***7.805***	***0.015***	0.860 (49)
	S	207	195	0.945	0.83	1.884	***0.050***	***7.055***	***0.205***	0.861 (46)

Statistically significant differences are highlighted in bold and italics. Endo = endosphere, Rhizo = rhizosphere, T = VWO-tolerant cultivars, S = VWO-susceptible cultivars, R^2^ = R^2^ of power law, St = Spearman’s *Rho* similarity threshold, avgK = average degree, avgCC = average clustering coefficient, GD = geodesic distance, Trans = transitivity, M = modularity, in brackets the number of modules

### Gene expression and key biological processes in olive cultivars tolerant and susceptible to *Verticillium dahliae*

**Fig. 4:**
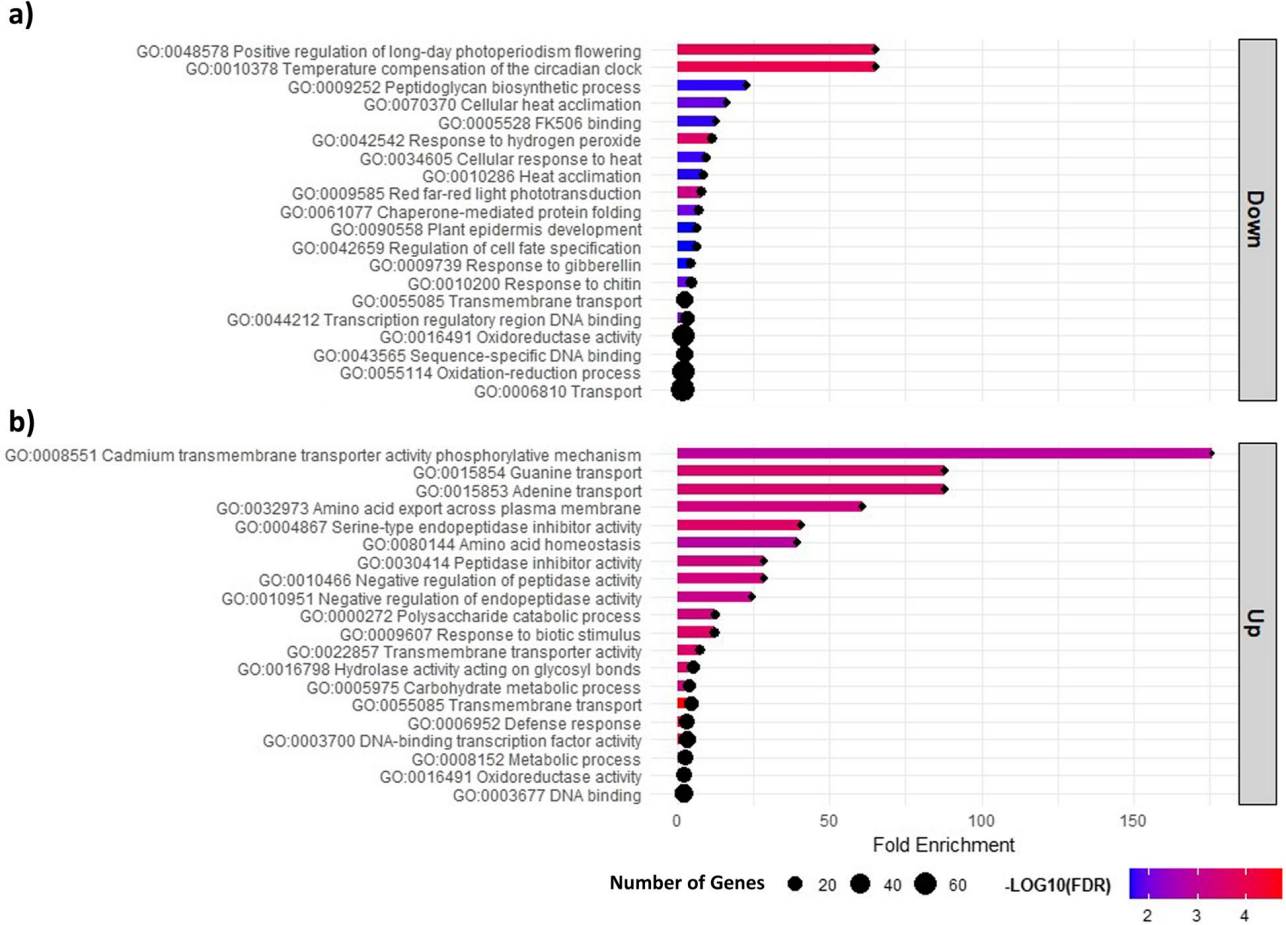
Biological processes highlighted in the gene enrichment analysis of differentially expressed genes. **a** The top 20 biological processes related to the overexpressed genes in VWO-susceptible cultivars. **b** The top 20 biological processes related to the overexpressed genes in VWO-tolerant cultivars

The transcriptome analysis revealed a total of 1,143 DEGs (*p*-values FDR corrected ≤ 0.05, –2 ≤ FC ≥ 2) when VWO-tolerant versus VWO-susceptible cultivars were compared. Out of the total, 309 were up-regulated in tolerant cultivars, whereas 834 were down-regulated, i.e., overexpressed in the susceptible cultivars. The enrichment analysis of both sets of DEGs revealed the most representative biological processes in each group of cultivars (Fig. 4).

Notable biological processes essential for maintaining cellular functions [e.g., transport of substances across the cell membrane (GO:0008551, GO:0015853, GO:0015854, GO:0022857, GO:0055085) and the specific transport and regulation of amino acids (GO:0032973, GO:0080144)] were found among the 309 genes overexpressed in VWO-tolerant cultivars (Fig. 4b). Terms related to the regulation of gene transcription (GO:0003700, GO:0003677) were identified as well. Genes involved in the metabolism and degradation of carbohydrates and polysaccharides (GO:0000272, GO:0005975, GO:0008152, GO:0016798), which are necessary for energy production, were also identified. Several terms related to the inhibition of peptidases and endopeptidases which may be preventing the degradation of proteins and peptides were also highlighted (GO:0004867, GO:0010466, GO:0030414, GO:0010951). Finally, genes coding for oxidoreductase activity (GO:0016491) and genes involved in defense response and response to biotic stimulus (GO:0006952, GO:0009607) were noted.

Concerning the VWO-susceptible group, genes associated with molecular transport (GO:0055085, GO: 0006810), transcriptional regulation (GO:0044212, GO:0043565) and regulation of cellular processes (GO:0042659, GO:0090558, GO:0009739) stood out among the 834 overexpressed genes (Fig. 4a). Remarkably, however, genes linked to stress response were prominent in the relevant biological processes found for this group of cultivars. Furthermore, the variability in terms related to stress responses was noteworthy, including those related to abiotic (GO:0010378, GO:0070370, GO:0010286, GO:0034605, GO:0048578, GO:0009585) and biotic (GO:09010200, GO:0009252) stress, as well as terms specifically related to cellular damage and oxidative stress responses (GO:0042542, GO:0016491, GO:0055114). GO:0005528 and GO:0061077 terms are also indicative of stress responses.

The identification of genes involved in plant defense and fungus response was performed by DEGs reannotation (See Supplementary_DEG_annotation sheet, Additional File 4) and by analyzing genes included in each biological process highlighted in the above-mentioned functional enrichment (See Supplementary_GeneEnrichment sheet, Additional File 4). Thus, in the group of genes overexpressed in the VWO-tolerant cultivars, a total of 57 genes were defense-specific or specific to fungal response. Similarly, in the group of genes overexpressed in VWO-susceptible cultivars, a total of 117 genes were related to plant defense and fungal response.

### Host plant-root microbiota interplay in the olive holobiont

The co-occurrence analysis between microbial endophytes (614 bacterial and 312 fungal ASVs) and olive DEGs (1,143) in the VWO-tolerant and VWO-susceptible cultivars revealed a strong correlation (Spearman's |*Rho*|≥ 0.73) among 32 bacterial and seven fungal ASVs with 300 DEGs [i.e., 97 up-regulated (Up) and 203 down-regulated (Down), taking the VWO-tolerant group as reference; See ‘Supplement ASV-DEG interaction’ sheet, Additional File 4]. Positive correlations were mostly linked to Up genes whereas negative correlations predominantly linked to Down genes. Most genes correlated with a single ASV. However, 87 DEGs correlated with several (from two to nine) ASVs. For instance, a BFN domain-containing protein (*bfn1*) and a SHSP domain-containing protein (*Hsp1*), correlated with seven and nine different ASVs, respectively.

**Fig. 5:**
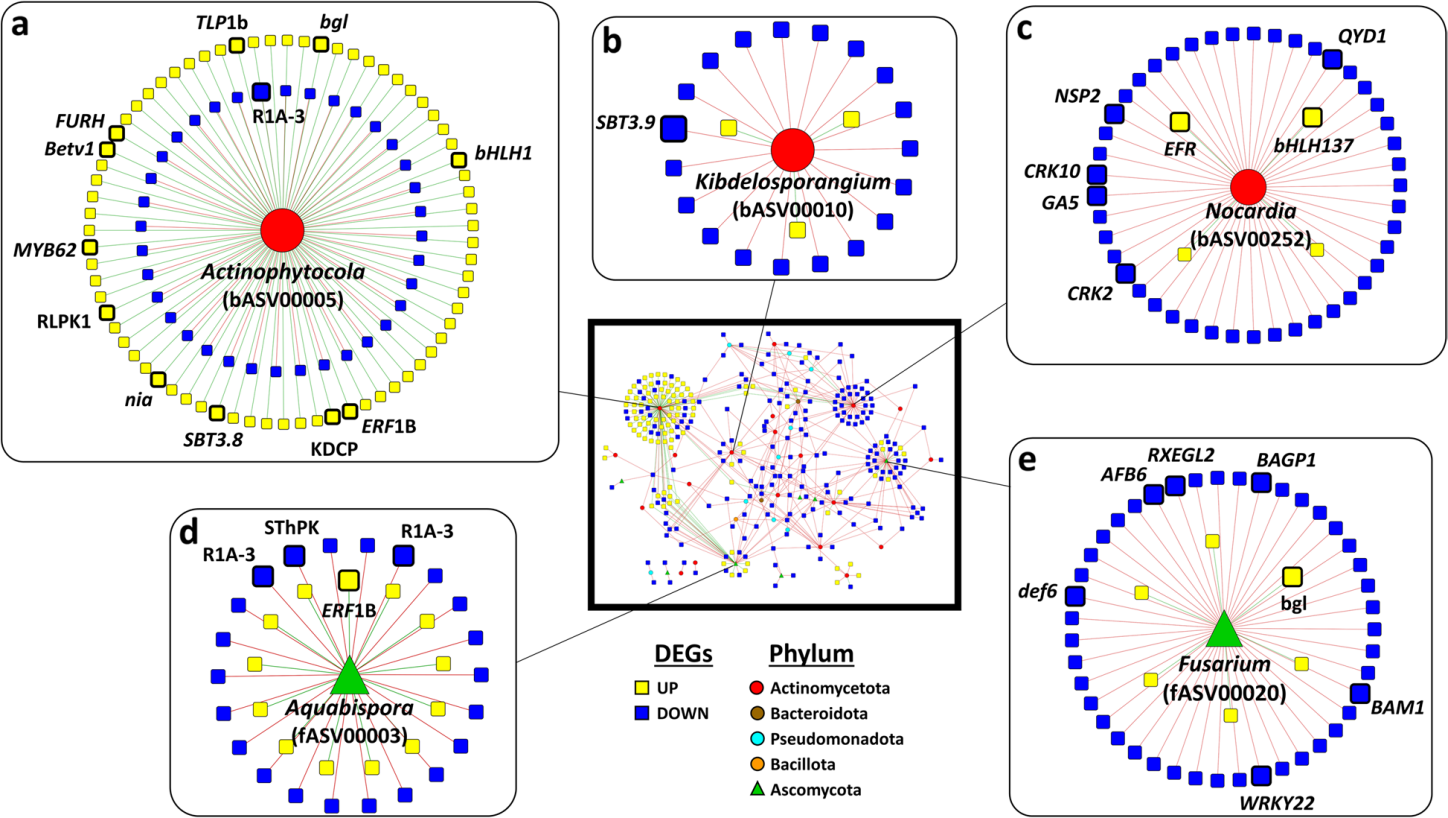
Co-occurrence network of microbial ASVs and differential expressed genes (DEGs). The whole network is in the central frame of the figure. Only ASV-DEG links were retained. The ASVs with the highest number of interactions were zoomed in. DEGs were considered as Up (yellow) or Down (blue) taking VWO-tolerant cultivars group as reference. *RLPK1* = Receptor-like protein kinase 1*, ERF* = Ethylene-responsive transcription factor*, KDCP* = Kinase domain-containing protein*, SThPK* = Non-specific serine/threonine protein kinase*, R1A-3* = Late blight resistance homolog *R1A-3, TLP1b* = Thaumatin-like protein 1b*, bgl* = β−1,3-glucanase*, FURH* = thioglucosidase-coding gene*, nia* = nitrate reductase*, RXEGL* = LRR receptor-like kinase*, def6* = defensin-like protein 6*, AFB6* = F-box protein 6*, CRK* = Cysteine-rich receptor-like protein kinase*, GA5* = ent-kaurene synthase/gibberellin-44 dioxygenase*, NSP2* = nodulation-signaling pathway 2 protein-like*, SBT* = subtilase-like proteins*, BAGP1* = BAG-associated GRAM protein 1, *Betv1* = major allergen *Bet* v1, *BAM1* = β-amylase, *bHLH* = bHLH domain-containing protein, *QYD1* = DELLA proteins RGL1-like, *MYB62* and *WRKY22* = defense response-related transcription factors

Out of the 39 microbial ASVs from the root endosphere showing correlation with the 300 olive DEGs, three bacterial ASVs (*Actinophytocola*, *Kibdelosporangium* and *Nocardia*) and two fungal ASVs (*Aquabispora* and *Fusarium*) stood out for being correlated with a high number of genes (Fig. 5). First, bASV00005, which is the most abundant ASV of the main genus (*Actinophytocola*), also correlated with the highest number of DEGs (107 genes; Fig. 5a). Among these genes, 12 were specifically related to plant defense and fungal response. Most of them corresponded to overexpressed genes in VWO-tolerant cultivars: a transcription factor (*MYB62*), a subtilisin-like protease (*SBT3.8*)*,* a thioglucosidase-coding gene (*FURH*), a kinase domain-containing protein, a receptor-like protein kinase 1, an ethylene (ET)-responsive transcription factor 1B-like, the thaumatin-like protein 1b, *Betv1*, a bHLH domain-containing protein (*bHLH1*), a β−1,3-glucanase (*bgl*), and a nitrate reductase (*nia*). Only the late blight resistance homolog *R1A-3* gene, which is down regulated in tolerant cultivars, was negatively correlated with bASV00005.

A second bacterial ASV correlating with a high number of DEGs belonged to the genus *Nocardia* (bASV00252). This ASV correlated with 45 Down and four Up genes. Among them, two positive correlations with overexpressed genes in VWO-tolerant cultivars, namely a transcription factor *bHLH137* and a *LRR* receptor-like serine/threonine-protein kinase *EFR*, were related to defense response. In addition, negative correlation was found with five down-regulated genes related to fungal defense response and symbiotic interactions (i.e., two Cysteine-rich receptor-like protein kinase (*CRK2* and *CRK10*), an ent-kaurene synthase/gibberellin-44 dioxygenase (*GA5*), a nodulation-signaling pathway (*NSP2*), and a *DELLA* protein *RGL1*-Like (*QYD1*) (Fig. 5c).

The third bacteria associated with a large number of DEGs belonged to genus *Kibdelosporangium*. Among the 22 genes correlating with this ASV (bASV00010), only one was related to defense response, a Subtilisin-like protease (*SBT3.9*)*,* which was down regulated in VWO-tolerant cultivars (Fig. 5b)*.*

Regarding to fungi, two ASVs were highlighted: one ASV (fASV00020; genus *Fusarium*) and another one belonging to *Aquabispora* (fASV00003), correlating with 49 and 35 DEGs, respectively. The *Fusarium* ASV correlated with seven defense-related genes, but only the glucan endo-1,3-β-glucosidase-coding gene *(bgl*) was up-regulated in VWO-tolerant cultivars. The negative correlations correspond to the following Down genes: *BAM1*, F-box protein (*AFB6*), LRR receptor-like kinase (*RXEGL2*), defensin-like protein (*def6*), *WRKY22* and *BAGP1* (Fig. 5e). The *Aquabispora* ASV correlated with four defense genes. This ASV showed positive correlation with the up-regulated gene encoding an ET-responsive transcription factor 1B-like (*ERF1B*)*,* and negative correlation with three down-regulated genes, two of them annotated as late blight resistance homolog (*R1A-3*), and one coding for a non-specific serine/threonine protein kinase (Fig. 5d)*.*

Additionally, other ASVs correlated with defense related genes. For instance, different ASVs belonging to *Actinophytocola* (bASV00012 and bASV00067) negatively correlated with Down genes *BAM1*, *WRKY22*, and a vacuolar sorting-associated protein 62. A *Parviterribacter* ASV was positively correlated with the Up gene encoding a Kunitz trypsin inhibitor 5 (*KTI5*). ASVs negatively correlating with Down defense-related genes in VWO-tolerant cultivars were *Pseudonocardia* (bASV00013), *Rhodomicrobium* (bASV00076), *Sphingomonas* (bASV00085) and *Peribacillus* (bASV00214). The corresponding genes were a *NIM1*-interacting 1-like, an argonaute-4, *NIK3*, and *ERF4* (See ‘Supplement ASV-DEG interaction’ sheet, Additional File 4).

## Discussion

The phenotypic trait ‘tolerance/susceptibility to VWO’ enabled to significantly discerning the belowground microbial profiles of olive cultivars displaying the most extreme phenotypes. This was possible even though this factor had moderate weight in the total variance explained by the distribution of the microbial profiles of the two groups of cultivars (i.e., VWO-tolerant and VWO-susceptible). In fact, cv. 'Maarri', of which only one published study evaluating its tolerance to VWO is available to our knowledge [24], showed microbial and transcriptomic profiles characteristic of the VWO-susceptible group (See Figure S2, Additional File 2). These authors reported that the number of asymptomatic ‘Maarri’ plants at the end of the bioassay was much lower than that scored for other VWO-tolerant genotypes [24]. Therefore, this observation underscores the significant complexity faced when qualifying the level of resistance/tolerance to Verticillium wilt in olive. While the study by Serrano and colleagues provides a general classification of several cultivars assessed under controlled and uniform conditions, it remains crucial to examine each case in detail, carefully considering the different parameters associated with the disease. Furthermore, it is imperative to include the influence of environmental factors and the interaction with the indigenous belowground microbiota in future experiments. Our results show that the combined analysis of both root microbiota and host transcriptome profiles is a holistic approach with great potential to evaluate olive genotypes under field conditions. Moreover, it can also serve as a complementary and powerful strategy to go beyond the traditional schemes used in olive breeding programs aiming to generate new VWO-tolerant/resistant varieties [48, 49]. That is, considering the crucial role played by the plant microbiome within the holobiont conceptual framework [8].

We previously described that actinobacteria are the predominant bacterial endophytes in olive roots [22]. This outcome was not surprising as these microorganisms are well adapted to the water-deficient conditions usually found in the Mediterranean region. Moreover, some genera (e.g., *Streptomyces*) have antimicrobial capacities and provide benefits to host plants [50, 51]. In the present study we identified a clearly different profile in the VWO-tolerant group which was mostly enriched in actinobacteria, *Actinophytocola*, *Kibdelosporangium* and *Nocardia* being the most abundant genera. These genera have been isolated from roots or rhizosphere soils of various plants. Some species of *Kibdelosporangium* and *Nocardia* have been reported to provide benefits to their hosts [52–54]. The potential benefit conferred to olive by *Actinophytocola*, the main genus found in our study, still needs to be confirmed experimentally. With regard to the endophytic mycobiome, an enrichment of *Fusarium* and *Aquabispora* was observed. The latter was classified in our previous work [22] as *Canalisporium* (another genus of the same family, *Savoryellaceae*). Both phytopathogenic [55] and beneficial [56] species of *Fusarium* are well known. However, to our knowledge, no studies have been carried out regarding the interaction of members of the *Savoryellaceae* family with plants beyond their role as organic matter decomposers [57]. Furthermore, enrichment in the rhizosphere of genera with plant beneficial species such as *Trichoderma* [58] and *Malassezia* [59] was also found.

Regarding the VWO-susceptible group, a higher proportion of the actinobacterial genera *Streptomyces* and *Pseudonocardia* together with *Bradyrhizobium* and *Pseudomonas* (phylum Pseudomonadota), and that of the arbuscular mycorrhizal fungal (AMF) genera *Rhizophagus* and *Dominikia*, were identified. An enrichment of these AMF in addition to *Septoglomus* was also found in the rhizosphere of this group of cultivars. It should be noted that all of them contain species beneficial to different plant holobionts [21, 51, 60–62]. Therefore, and considering that the genotype is a key factor in shaping the olive root-associated microbiota [22], we conclude that the two groups of cultivars here examined have different strategies to recruit, assemble and co-evolve with their microbial community.

Co-occurrence network analyses revealed that members of the microbial communities in each cultivar group interacted with each other in clearly different ways. They also showed very different keystone taxa, being more pronounced in the root endosphere than in the rhizosphere. Remarkably, ASVs of the genera *Actinophytocola*, *Virgisporangium* and *Cryptosporangium* stood out in the co-occurrence network of the endophytic microbiota of the VWO-tolerant group since they negatively correlated with the ASV identified as *Verticillium*. In our previous study, a positive correlation of *Actinophytocola* with expression levels of olive genes related to defense and antifungal activity was found [15]. Regarding the poorly-investigated actinobacterium *Virgisporangium* (family *Micromonosporaceae*), little can be said about its role in the olive belowground microbiome. However, a comparative genomics study has predicted the production by this genus of dynaplanins, a family of compounds with antimicrobial activity, what highlights its potential as a biocontrol agent [63]. The same can be argued for the so-called rare actinomycetes *Cryptosporangium* (poorly-investigated too), that produces antimicrobial compounds known as wychimicins [64]. The endophyte microbiome co-occurrence network of the VWO-susceptible group showed more heterogeneous keystone taxa than the endophytome of VWO-tolerant cultivars. It is noteworthy that the endophyte mycobiome of VWO-susceptible cultivars seemed to play a more relevant role in the community assembly, at least some of its constituents. Indeed, one ASV of the genus *Penicillium* was identified as network hub. Species of this genus have been demonstrated as potent biocontrol agent against different plant pathogenic fungi [65]. However, even though this ASV being essential in the assembly of the endophytic fungal community, no correlation with plant defense response genes was found. In the case of AMF, their beneficial interaction with the plants is well known [66]. In this present study, however, the interactions unveiled with other members of the olive belowground microbiota, notably those of the genus *Rhizophagus*, are suggestive of additional and potentially crucial roles. Therefore, they do not only provide direct benefits because of the symbiosis established with the host, but also could contribute (indirectly) in a defensive barrier by interacting negatively with ASVs of phytopathogenic genera (e.g., *Macrophomina* and *Agrobacterium*) (Fig. 3a). In fact, the role of AMF as biocontrol agents has been previously demonstrated [67].

Regarding the macroscopic component of the olive holobiont, our results are in line with a previous study describing the differential basal genetic defense response against *V. dahliae* observed among olive cultivars [23]. It is worth noting that we observed a higher number of defense-related genes overexpressed in the roots of VWO-susceptible cultivars than in the VWO-tolerant ones. Remarkably, the genes overexpressed in the latter group were mainly involved in the activation of defense mechanism, whereas genes overexpressed in cultivars susceptible to *V. dahliae* were mostly related to stress and damage response (Fig. 4b). Accordingly, the inhibition of peptidase activity, enriched in VWO-tolerant cultivars, has been linked to a protective reaction of plants suppressing the growth of fungal mycelium [68]. Several genes enriching the biological process of defense response in these cultivars [e.g., ET-responsive transcription factors (*ERF*), salicylic acid (SA) binding protein (*SABP2*), exopolysaccharide receptor (*EPR*), thaumatin-like proteins (*TLP*), *MYB* genes, PR10 or major pollen allergen (*Betv1*), and β−1,3-glucanases and chitinases enzymes; See Supplementary_GeneEnrichment sheet, Additional File 4], and that were overexpressed in this group, could hamper the infection by *V. dahliae* or attenuate VWO symptoms development [69–73]. Interestingly, chitinases and PR10 along with β−1,3-glucanase reduced VWO symptoms in the tolerant olive cultivar ‘Sayali’ [69]. Furthermore, *PR2* genes, coding for β−1,3-glucanase*,* were up-regulated in VWO-tolerant olive cultivars upon *V. dahliae* inoculation [26]. Finally, *TLP* genes have been related to resistance towards *V. dahliae* due to their role in inhibiting hyphal growth and increasing secondary cell wall thickening in olive [69, 74].

Concerning VWO-susceptible cultivars, genes involved in heat stress response highlighted enriching several biological processes, including response to hydrogen peroxide. In this line, it has been observed that the susceptibility to verticillium wilt could increase when plants are subjected to high temperature [75]. Nonetheless, VWO symptoms are similar to those caused by water or heat stress [76], so the response mechanism may be common. For instance, GIGANTEA proteins and heat stress transcription factors stood out enriching biological processes in susceptible cultivars. The *GIGANTEA* gene plays a fundamental role in the response to abiotic stresses like freezing, salinity, drought and osmotic stress [77]. Supporting this hypothesis, some heat shock factors (HSFs) are also involved in the response against *V. dahliae* throughout the synthesis of secondary metabolites [78].

We demonstrated that both the belowground microbial communities and the gene expression pattern of olive roots showed sharp differences between VWO-tolerant and VWO-susceptible cultivars. Besides noting this fact, to unravel links between the endosphere microbiota (or some of its constituents) and host gene expression at the root level would be of utmost relevance. Indeed, the microbial communities present in the olive root endosphere, as a whole, could play a determining role in the activation of defense mechanisms against *V. dahliae*. Moreover, the up or down regulation of certain host genes may also decisively influence the composition of root microbial communities [79]. By implementing the strategy designed in our study, several ASVs were found to correlate with olive DEGs, including genes involved in plant defense responses. Among these ASVs, one from *Actinophytocola* and another one from *Nocardia* are worth mentioning since both of them were significantly more abundant in VWO-tolerant cultivars.

The genus *Actinophytocola* correlated negatively with *Verticillium*, and one *Actinophytocola* ASV (i.e., bASV00005) showed positive correlation with defense-related genes previously reported to be involved in defense against *V. dahliae*. For instance, *MYB* genes were reported to modify root architecture and enhance the resistance to water stress and verticillium wilt [80, 81]. It is tempting to speculate that *Actinophytocola* could be somehow involved in the activation of these genes in olive roots. Other genes putatively involved in defense against VWO positively correlated with this actinobacterium as well. This was the case of *bHLH1,* a gene previously reported to be induced by *Pseudomonas simiae* PICF7 [82], an effective biocontrol agent of VWO and an indigenous endophyte of olive roots [83, 84]. Another defense-related gene associated with *Actinophytocola* was the subtilase *SBT3.8*. Subtilase-coding genes have been further related to the induced systemic resistance mechanism against *V. dahliae* in cotton [85]. Similarly, bASV00005 positively correlated with a β−1,3-glucanase-coding gene. The use of bacteria releasing β−1,3-glucanase has been proposed as a biocontrol strategy [86]. On the one hand, our results suggest that *Actinophytocola* could not only be a key player in structuring the olive root endophytome, but also in the microbiome-host transcriptome dialogue occurring within the roots of the olive holobiont. This olive genes-endophytic *Actinophytocola* sp. interplay would contribute to explain VWO-tolerance, as it was earlier suggested for a different (abiotic) stressor [15]. On the other hand, and for an agrobiothecnological point of view, *Actinophytocola* could have great potential as a plant growth promotion rhizobacterium, in general, and as biocontrol agent against *V. dahliae* in particular.

The negative correlation found between an ASV from the genus *Nocardia* with fungal defense-related genes repressed in VWO-tolerant cultivars could suggest a symbiotic interaction with the olive roots. Genes involved in these interactions are related to the gibberellins (GAs), like ent-kaurene synthase/gibberellin-44 dioxygenase (*GA5*)*, DELLA* proteins *RGL1*-like (*QYD1*) and nodulation-signaling pathway 2 protein-like (*NSP2*). These genes together with *LRR-EFR* genes (positively correlated with *Nocardia*) were reported to promote nodule formation in legumes by means of reducing plant defense mechanisms [87]. The *gibberellin-44 dioxygenase* gene is related to the biosynthesis of terpenoids, which have antifungal activity [88]. These findings may support the role of some species of *Nocardia* in the immune response against *V. dahliae* in tolerant cultivars. Therefore, *Nocardia* might play a dual role within the olive root endophytome. By influencing phytohormone pathways and potentially enhancing antifungal defenses, this genus could contribute to both plant-microbiome communication and stress resilience of the olive holobiont.

## Conclusions

**Fig. 6:**
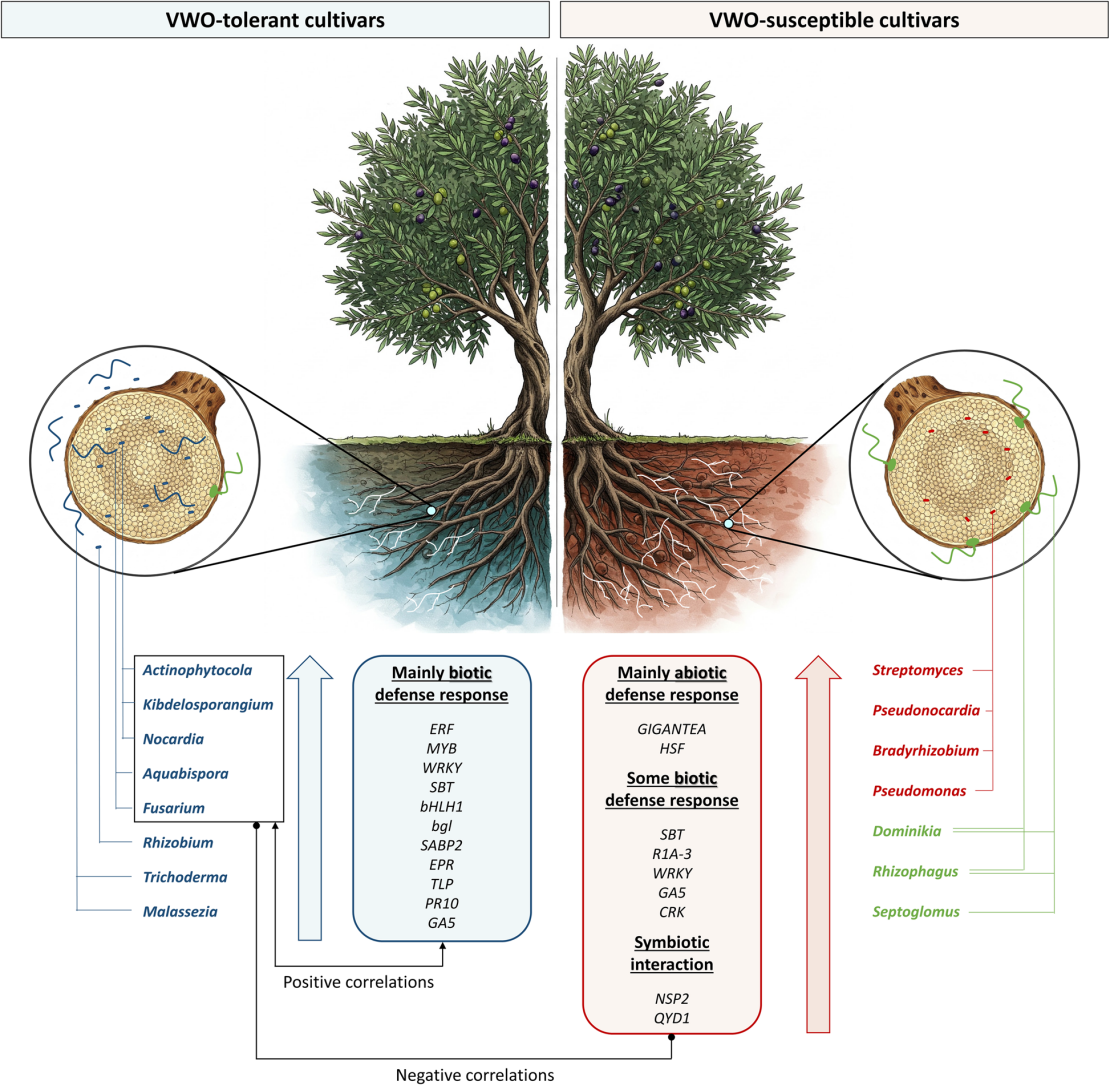
Schematic figure summarizing the main differences at the belowground microbiome and root host transcriptome level. Taxa framed in the black box refer to those highlighted in Fig. 5. *ERF* = Ethylene-responsive transcription factor*, MYB* and *WRKY* = defense response-related transcription factors, *bHLH* = bHLH domain-containing protein, *EPR* = exopolysaccharide receptor, PR10 or major pollen allergen (*Betv1*), *R1A-3* = Late blight resistance homolog *R1A-3, TLP* = Thaumatin-like protein*, bgl* = β−1,3-glucanase*, CRK* = Cysteine-rich receptor-like protein kinase*, GA5* = ent-kaurene synthase/gibberellin-44 dioxygenase*, NSP2* = nodulation-signaling pathway 2 protein-like*, HSF* = heat shock factors*, SBT* = subtilase-like proteins*, SABP2* = salicylic acid binding protein 2*, QYD1* = DELLA proteins RGL1-like. The olive tree image was generated with Gemini 2.0 Flash on March 27th, 2025. https://gemini.google.com/app?hl=es-ES

This study underscores the importance of identifying keystone taxa along with essential host plant genes to understand plant-microbiota interactions and explore their potential in disease management. Indeed, this integrative approach provides insights into the complex interplay taking place between plants and their microbiota, offering potential targets for microbiome engineering to enhance olive resilience against VWO. Overall, our results suggest that *Actinophytocola* and *Nocardia* could be crucial in the microbiome-host transcriptome dialogue occurring within the roots of the olive holobiont (Fig. 6). The methodological approach here implemented also shed light on how the micro- and macroscopic components of the olive holobiont might have coevolved to explain differences in tolerance/susceptibility to VWO at the subspecies level (i.e., cultivars). Furthermore, we emphasize the importance of isolating and in-depth characterizing keystone taxa in order to experimentally demonstrate the functions here envisaged. To unravel whether: (i) microbial keystones are decisively modulating the olive gene expression to overcome *V. dahliae* infection at the root level; (ii) the host genotype fails or succeeds in shaping a root microbiota able to confront *V. dahliae* attack; or (iii) only an effective dialogue between both components of the olive holobiont is the determining factor to explain the phenotype here examined still need experimental evidence. However, the identification of: (i) relevant taxa present in the indigenous olive root microbiota linked to tolerance or susceptibility to VWO; (ii) host genes involved in a range of defense responses that are up/down regulated depending on the VWO tolerance level; and, most importantly, (iii) the correlation found between the expression of specific host genes and particular constituents of the olive belowground microbiota can now pave the way to answer the questions mentioned above.

## Supplementary Information

Additional File 1. Excel document in xlsx format. It contains three sheets. The first one is the bacterial ASV table (Bacterial_ASV_final). The second one is the fungal ASV table (Fungal_ASV_final). The third one corresponds to the soil and root samples metadata (country of origin, cultivar name, sample department (endo- or rhizosphere), VWO-susceptibility classification according to Serrano et al. [24], final selection of cultivars compared in the present study) (Samples_metadata).

Additional File 2. Word document in docx format. It contains Table S1, Figure S1 and S2.

Additional File 3. Tables with the relative abundance at genus level of bacterial and fungal communities in both endosphere and rhizosphere. Excel document in xlsx format. It contains four sheets. Genera with relative abundance ≥ 1 are highlighted in light green and ≥ 2 in dark green. Colored genus names are highlighted according to their differential abundance when comparing VWO-tolerant and VWO-susceptible cultivars only with ANCOM-BCII, ALDEx2 or with both methods.

Additional File 4. Tables of gene annotation and interaction with ASVs. Excel document in xlsx format. It contains seven sheets. The first sheet shows the gene enrichment of all the DEGs found in this study. It also has the p-values FDR corrected, number of genes of each pathway, fold enrichment and the Gene Ontology annotation. The second sheet contains the 1,143 DEGs together with their Log2FoldChange, p-value and annotation. The third one shows all the ASVs and DEGs contained in the whole network from Figure 5. The five ASVs from that figure and the ‘Plant defense response’ related genes are highlighted in bold. The last four sheets, “nodes sheets”, (Endo_T_nodes, Endo_S_nodes, Rhizo_T_nodes and Rhizo_S_nodes) contain the main parameters of all nodes in each network. ‘Endo’: endosphere, ‘Rhizo’: rhizosphere, ‘T’: VWO-tolerant cultivars, and ‘S’: VWO-susceptible cultivars. Keystone (network hubs, module hubs and connectors) are highlighted.

## Data Availability

The datasets analyzed during the current study are available in the NCBI Sequence Read Archive (SRA) under the BioProject number PRJNA498945 (olive belowground microbiota), and in the Gene Expression Omnibus (GEO) repository, accession number GSE152236 (root plant RNA-Seq).
