# Supplementary material for: Unveiling essential host genes and keystone microorganisms of the olive tree holobiont linked to Verticillium wilt tolerance
Source: Microbiome. 2025 Nov 26;13:239. doi: 10.1186/s40168-025-02216-5 (PMC12659499; doi:10.1186/s40168-025-02216-5)
Supplement: Supplementary file 3 — Additional File 2. Word document in docx format. It contains Table S1, Figure S1 and S2. [file 40168_2025_2216_MOESM2_ESM.docx]

**Table S1. Initial analysis of alpha and beta diversity of olive groups tolerant and susceptible to VWO.**

Statistically significant *p*-values are highlighted in bold type and italics.


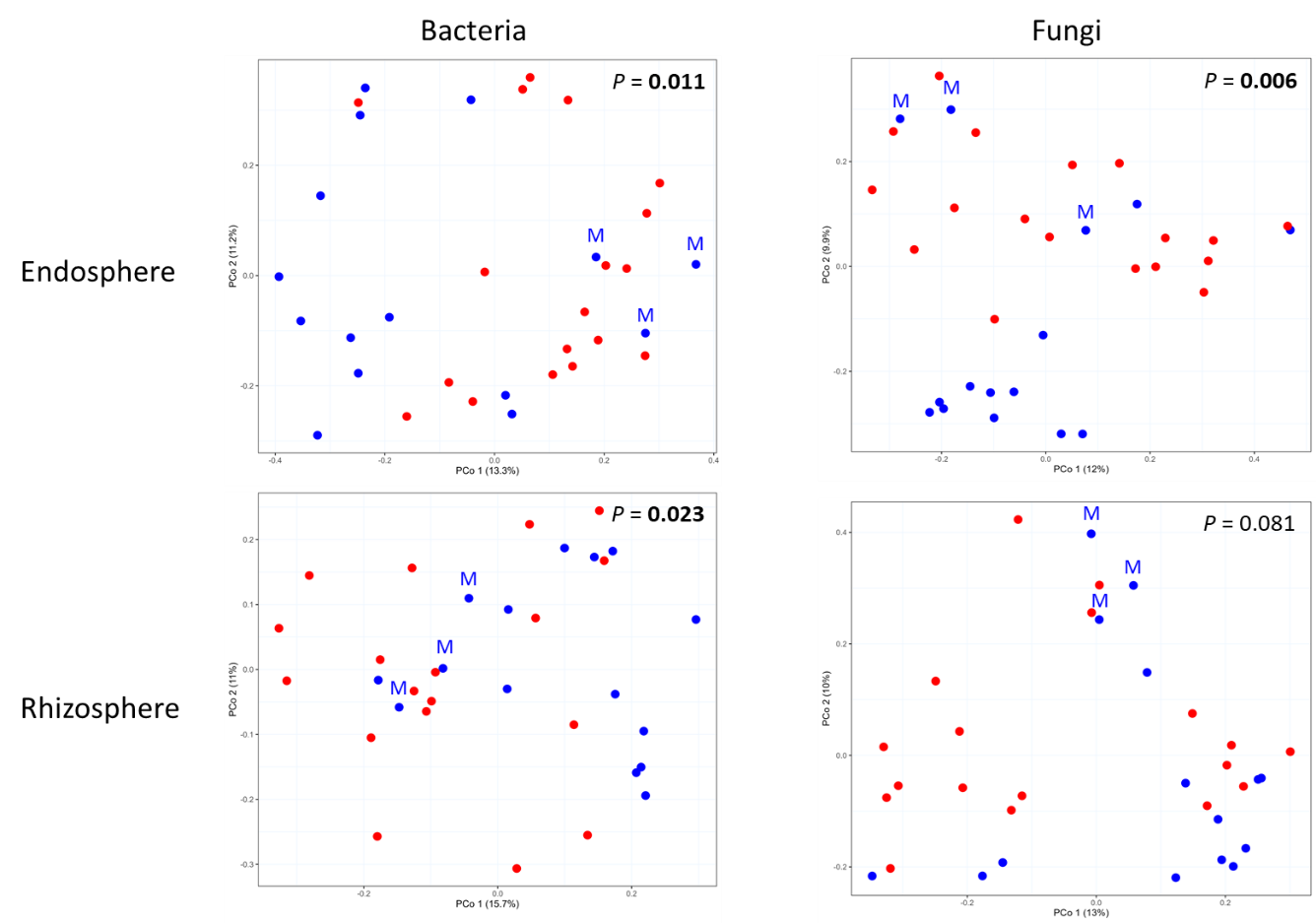


**Figure S1.** **Principal Coordinate Analisis (PCoA) of belowground microbial communities.**

VWO-tolerant cultivars are shown in blue and VWO-susceptible cultivars in red. M = cultivar ‘Maarri’. Statistically significant *p*-values of PERMANOVA test are highlighted in bold type and italics.


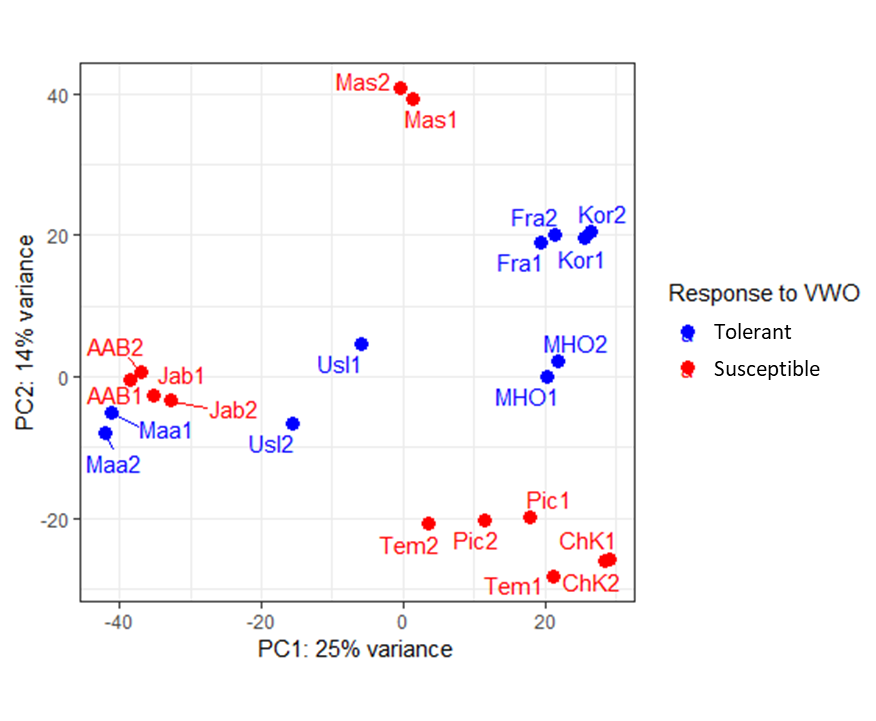
**Figure S2. Principal Component Analysis (PCA) for host transcriptomic response.** VWO-tolerant cultivars are shown in blue and VWO-susceptible cultivars in red. Two trees (replicates) for each cultivar. AAB =Abbadi Abou Gabra-842, Jab = Jabali, Maa = Maarri, Usl =Uslu, Mas = Mastoidis, Tem = Temprano, Pic = Picual, Fra = Frantoio, Kor = Koroneiki, MHO = Manzanillera de Huércal-Overa, ChK = Chemlal de Kabylie.
